# Protective Effects of Rapamycin on Trabecular Meshwork Cells in Glucocorticoid-Induced Glaucoma Mice

**DOI:** 10.3389/fphar.2020.01006

**Published:** 2020-07-02

**Authors:** Xiaolu Zhu, Shengyu Wu, Wen Zeng, Xiaomin Chen, Tian Zheng, Jiangbo Ren, Min Ke

**Affiliations:** Department of Ophthalmology, Zhongnan Hospital of Wuhan University, Wuhan, China

**Keywords:** glucocorticoid-induced glaucoma, trabecular meshwork, dexamethasone, autophagy, rapamycin

## Abstract

Glucocorticoid-induced glaucoma (GIG) is a chronic optic neuropathy caused by systemic or topical glucocorticoid (GC) treatment, which could eventually lead to permanent vision loss. To investigate the protective effects of rapamycin (RAP) on the trabecular cells during the development of GIG in mice, the effects of RAP on intraocular pressure (IOP), trabecular ultrastructure, and retinal ganglion cells (RGCs) were examined in C57BL/6J female mice treated with dexamethasone acetate (Dex-Ace). The expression of α-actin in trabecular tissue was detected by immunofluorescence, and the autophagic activity of trabecular cells and the expression of GIG-related myocilin and α-actin were detected by immunoblotting. Our results indicated that Dex-Ace significantly increased IOP at the end of the third week (*p* < 0.05), while RAP treatment neutralized this elevation of IOP by Dex-Ace. Dex-Ace treatment significantly decreased the RGC numbers (p < 0.05), while synchronous RAP treatment kept the number comparable to control. The outer sheath of elastic fibers became thicker and denser, and the mitochondria of lesions increased in Dex-Ace-treated groups at 4 weeks, while no significant change was observed in the RAP-treated trabecular tissues. Dex-Ace induced myocilin, α-actin, Beclin-1, and LC3-II/LC-I ratio, and lowered p62, while synchronous RAP treatment further activated autophagy and neutralized the induction of myocilin and α-actin. Our studies suggested that RAP protected trabecular meshwork cells by further inducing autophagy way from damages of GC treatment.

## Introduction

The therapeutic use of glucocorticoids (GCs) in susceptible individuals increases intraocular pressure (IOP) (Fini et al., 2017), which is a major risk factor for GC-induced glaucoma (GIG), an ocular diseases featured with progressive degeneration of retinal ganglion cells (RGCs) (Kwon et al., 2009). In addition, GIG is similar to primary open angle glaucoma (POAG). Increased IOP can cause vascular insufficiency (Ster et al., 2014). and will further lead to vascular endothelial metabolic disorders (Gong et al., 2016a; Fu et al., 2018a). GC-induced ocular hypertension results from increased aqueous outflow pathway resistance, morphological and biochemical changes in trabecular meshwork (TM) (Clark and Wordinger, 2009). Therefore, the effects of GCs on TM cells and other ocular tissues drew increasing attention during last decades. Previous researches suggested that GCs promoted the deposition of TM extracellular matrices (such as fibronectin and type IV collagen), cytosolic protein (such as α-smooth muscle actin), and altered cell cytoskeleton to form cross-linked actin networks (Chan, 2006; Deng et al., 2013). However, the exact pathological mechanisms are still unclear at present. A comprehensive knowledge on the pathogenesis of steroid responders will improve our prevention of IOP elevation and enhance our understanding of steroid induction mechanisms in glaucoma.

Autophagy is an important process to promote cell survival under various stressful conditions, during which various stromal and organelles in the cell are degraded by the lysosomal system. TM cells belong to the post-dividing cells and cannot be removed by re-splitting to remove excess harmful substances. TM cells are continuously under mechanical stress and cell deformation stress due to IOP fluctuations and eye movement (Hirt and Liton, 2017). Autophagy, as a mechanism of intracellular self-adaptation protection, maintains TM cell homeostasis and normal function. Previous reviews stated that GCs affected autophagy of various cells, such as osteoblasts, fibroblasts, muscle cells, and lymphocytes (Eisenberg-Lerner et al., 2009; Zhu and Zhang, 2018). Researches also showed that the autophagy homeostasis of TM cells in the glaucoma aqueous humor outflow pathway changed (Eskelinen and Saftig, 2009). In primary cultures of porcine and human TM cells, sustained IOP elevation activated autophagy to response pressure and restore balance (Porter et al., 2014).

Our previous researches suggested that the dexamethasone acetate (Dex-Ace) treatment activated autophagy in a time-dependent manner and that the autophagy activity peaked at the fourth week with a plateau of increased IOP for 4 weeks. Thereafter, the continued DEX-Ace treatment did not affect IOP value reduction, but the autophagy activity gradually decreased (Zeng et al., 2019). Decreased autophagy activity might cause the accumulation of diseased organelles, and produce oxidative damage as well (Levine and Klionsky, 2004), and might be an indication of progressive dysregulation of TM function.

Rapamycin (RAP), a lipophilic macrolide antibiotic, was created as an antifungal agent, and also has multifunctional nonantibiotic properties (Prevel et al., 2013). Related researches showed that RAP played an important role in neurological diseases, like Parkinson’s disease (Malagelada et al., 2010), nerve injury, Alzheimer disease, and so on (Caccamo et al., 2009). Previous studies stated that RAP improved the survival rate of RGCs in a rat chronic ocular hypertension model of glaucoma (Su et al., 2014) and significantly enhanced autophagy in a monkey chronic hypertensive model (Deng et al., 2013). However, the role of RAP in GIG is still unclear.

Studies using systemically or topically treated C57BL/6J mice with DEX showed increased IOP and ultrastructural changes looked like those stated in humans after GC therapy (Overby et al., 2014; Zode et al., 2014; Patel et al., 2017; Faralli et al., 2018). This research, we used a GIG mouse model to explore the relationship between elevated levels of autophagy and hormonal glaucoma. The effects of RAP, as an mTOR inhibitor and autophagy inducer (Levine and Klionsky, 2004), on the autophagy levels in GIG mice were investigated. Our results indicated that RAP protected the functions of TM cells *via* upregulating autophagy in GIG.

## Materials and Methods

### Animal Experiment

Female C57BL/6J mice (6–8 weeks old) were purchased from Beijing HFK Bioscience company and housed at the Center for Animal Experiment/Animal Biosafety Level-III laboratory of Wuhan University. Animal experiment complied with the Association for Research in Vision and Ophthalmology Statement of the Use of Animals in Ophthalmic and Vision Research and carried out according to the regulation of Wuhan University Health Science Center Institutional Animal Care and Use Committee. The mice (16–18 g) were housed under a 12-h light/12-h dark cycle with a free access to standard rodent food and water. The condition of temperature was controlled (22–28°C), as well as the humidity (45–75%).

### Reagents

One hundred and six C57BL/6J mice were divided into four groups randomly: control (vehicle suspension +DMSO), Dex-Ace-treated (Dex-Ace+DMSO), RAP-treated (vehicle suspension +RAP), and Dex-Ace+RAP-treated groups (Dex-Ace+RAP). Dex-Ace (10 mg/ml) or vehicle suspension solution (20 μl) was conjunctival fornix (CF) injected into the tenon of the right eye every 4 d. RAP (4 mg/kg) or 0.1% dimethyl sulfoxide (DMSO, 100 μl) was injected intraperitoneally every other day. Vehicle suspension and DEX-Ace formulation were introduced as a preview study (Patel et al., 2017). RAP formulation (0.25 mg/ml in 0.1% DMSO) was stored at -20°C (working fluid).

### Periocular CF Injection

Mice were put into an anesthesia chamber filled with 0.8 L/min oxygen and 2.5% isoflurane to induce general anesthesia. After anesthesia, mice got 1–2 drops of 0.5% proparacaine HCL (S.A. Alcon-couvreur N.V., Belgium) in both eyes. The CF injection was performed as previously noted (Patel et al., 2017). Briefly, after lower eyelid was retracted, 20 μl DEX-Ace (10 mg/ml) or vehicle suspension were injected by a 29-gauge insulin syringe immediately under the CF of the right eye in the process of 5–10 s. A 1-ml syringe (Sinopharm, China) was used to inject 100 μl RAP (4 mg/kg) or DMSO (0.1%) into the abdominal cavity.

### IOP Measurement

Mice were put into the Decapicone, a plastic bag especially for the mouse, which could fully expose the head but restrict its movement (Wang et al., 2005). The head of conscious mice was exposed in the hole at the top of the cone and IOP was measured as soon as the mice stayed stable. An effective reading of daytime IOP was obtained weekly at 10 am and 2 pm by applying a probe of tonometer (TonoLab, Colonial Medical, USA) to gently flatten an area of the corneal surface. After baseline IOP was obtained, the right eye was then treated with DEX-Ace or vehicle suspension every 4 d. The IOP of each eye was taken from the average of 3 test values.

### Weight Measurement

The mice were gently placed on the digital electronic scales (BY-dzc, China) and the weight was measured immediately after the mice stayed stable. Recorded effective reading of body weight weekly at 10 am to an accuracy of 0.01 g. The body weight of each mice was taken from the average of three test values.

### RGC Staining

To estimate changes in the RGC numbers after GIG mice were induced, we counted the number of RGCs in the retinas. BRN3a were used to detect the RGCs in the retina, and the method of retina dissection was as previously described (Li et al., 2014; Vidal-Sanz et al., 2015; Wang et al., 2016; Gong et al., 2016b). Briefly, after the mice were sacrificed, the enucleated eyes were fixed in 4% paraformaldehyde for 1 h and flushed in PBS. The retinas were then cut into 4 quadrants and flattened with a fine brush. After incubated with 0.5% Triton X-100 for 15 min, the retinas were incubated with BRN3a antibody (1:200, Millipore, USA) at 4°C overnight. After incubating, the retinas were flushed in PBS three times and then incubated with IgG Cy3 antibody (1:200) for 2 h. Non-overlapping images containing most of the retina in four quadrants were obtained by confocal 155 microscope (zoom = 1,600 folds; TCS SP5 CLSM, Leica, Germany), and the average RGC numbers for four quadrants were quantified.

### Transmission Electron Microscopy

Unperfused mouse eyes were immediately fixed *via* 2.5% gluataraldehyde (Ted Pella, USA) in phosphate buffer at 4°C for 2 h. The fixative was injected into the eye from a tiny incision in the posterior sclera. Tissues were then fixed with OsO4, dehydrated using ascending alcohol series, and embedded in Epson resin. Ultrathin sections on trabecular organization were cut with an ultramicrotome (EM UC7, Leica), examined using an electron microscope (Tecnai G2 20 TWIN, FEI, USA), and then obtained as described previously (Zeng et al., 2019).

### Immunofluorescence, Hematoxylin and Eosin (H&E) Staining

Mouse eyeballs were enucleated and immediately fixed in 4% paraformaldehyde at 4°C overnight as previously described (Fu et al., 2018b). After rinsed three times with PBS, the eyes were dehydrated and embedded in paraffin (Paraplast, Sigma-Aldrich, USA). Tissue slices (5 μm) were obtained using a rotation microtome (Thermo Fisher, USA), deparaffinized, and then rehydrated with graded ethanol for 5 min twice each. Antigen retrieval was conducted in citrate buffer. Once cooled, tissue sections were blocked with 10% goat serum and 0.2% Triton-X 100 in a dark and humid chamber for 2 h. After rinse briefly with PBS, the sections were immunolabeled with rabbit polyclonal antibody (α-smooth muscle actin, 1:100, Abcam) and incubated at 4°C overnight. After flushing, the samples were incubated with corresponding secondary antibodies (Alexa goat anti-rabbit 568, 1:500, Thermo Fisher) for 2 h. DAPI (Vector, CA) was used to visualize cellular nuclear. The slices were examined by the Keyence all-in-one fluorescence microscope (Itasca, USA) (Kasetti et al., 2016). For H&E staining, the paraffin section of mice TM tissues were sequentially deparaffinized, rehydrated, stained with hematoxylin and eosin (Sigma-Aldrich), dehydrated and sealed. The slices were visualized and photographed with phase contrast microscope (DMI 1, Leica).

### Western Blotting

Anterior segment tissues were dissected detailedly and then placed in RIPA lysis buffer (Cell Signaling Technology, USA) (Zode et al., 2015). Whole section of the TM with small part of ciliary muscle, iris, and cornea, were contained in the tissues. The BCA Protein Assay kit (Beyotime, China) was used to detect the concentration of total protein. Total protein (40 μg) was analyzed by sodium dodecyl sulfate polyacrylamide gel electrophoresis (Beyotime) and transferred to polyvinylidene fluoride membranes (Millipore, USA) followed by the manufacturer’s protocol. Membranes were blocked by PBS containing 5% BSA (Cell Signaling) at room temperature for 1 h, and then incubated with primary antibodies (p62, 1:1,000, Cell Signaling; Beclin-1, 1:1,000, Abcam; α-actin, 1:1000, Abcam; Myocilin, 1:500, Abcam; GAPDH, 1:1000, Boster, China) at 4°C overnight. The membranes were washed, incubated with corresponding secondary antibodies (1:3,000, Cell Signaling), and detected by ChemiDocTM XRS+ Imaging System (Bio-Rad, USA). The band intensities were analyzed by ImageJ software.

### Statistical Analysis

Statistical analyses were performed with using Prism version 7.0 (GraphPad, USA). Statistical analyses among groups were evaluated *via* one-way analysis of variance (ANOVA), and two groups’ comparisons were using unpaired t-test. All data were presented as mean ± SEM for multiple independent experiments. *P* < 0.05 was considered as statistical significance.

## Results and Discussion

### RAP Inhibited the Elevated IOP Caused by Dex-Ace CF Injection

**Figure 1:**
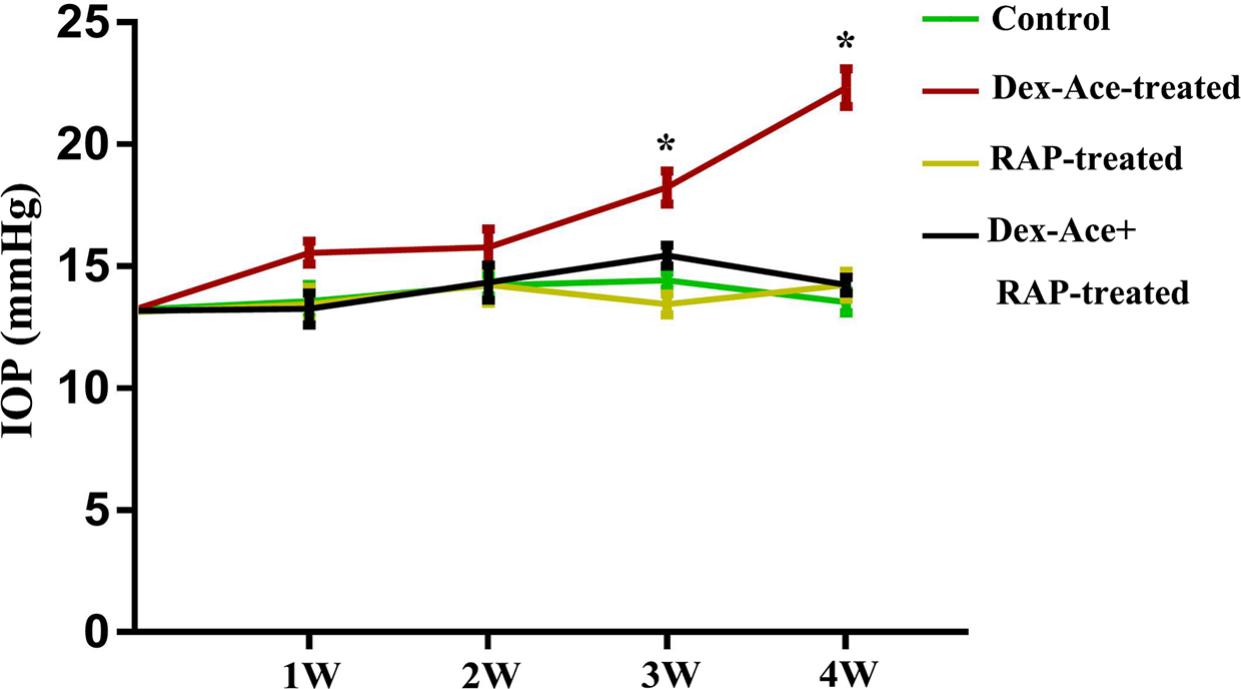
RAP reduced Dex-Ace-induced IOP elevation. The conscious mouse IOP was induced significantly by Dex-Ace at 3–4 weeks, but RAP treatment decreased this increase. (n = 20; *, *p* < 0.05).

The CF injection of Dex-Ace was reported to repeatedly caused obvious and persistent IOP elevation, which related to reduced outflow facility (Patel et al., 2017). As our previous researches proved, Dex-Ace induced rapid and significant IOP increase, which peaked at week 4 (Zeng et al., 2019). In this study, we founded that the IOP in the Dex-Ace-treated group increased at 3 weeks and sustained until 4 weeks. However, IOP had little change in the other three groups. Conscious daytime IOP value was 13.54 ± 0.45 mmHg (n = 18) in the control group, 22.33 ± 0.77 mmHg (n = 18) in the Dex-Ace-treated group, 14.23 ± 0.65 mmHg (n = 18) in the RAP-treated group, and 14.23 ± 0.68 mmHg (n = 18) in the DEX-Ace+RAP-treated group at week 4. Since the third week of treatment, the conscious mouse IOP in the DEX-Ace-treated group continued to be higher than the other three groups by about 4–8 mmHg (*P* < 0.05) (**Figure 1**).

### RAP Did Not Affect the Body Weight in Mice

**Figure 2:**
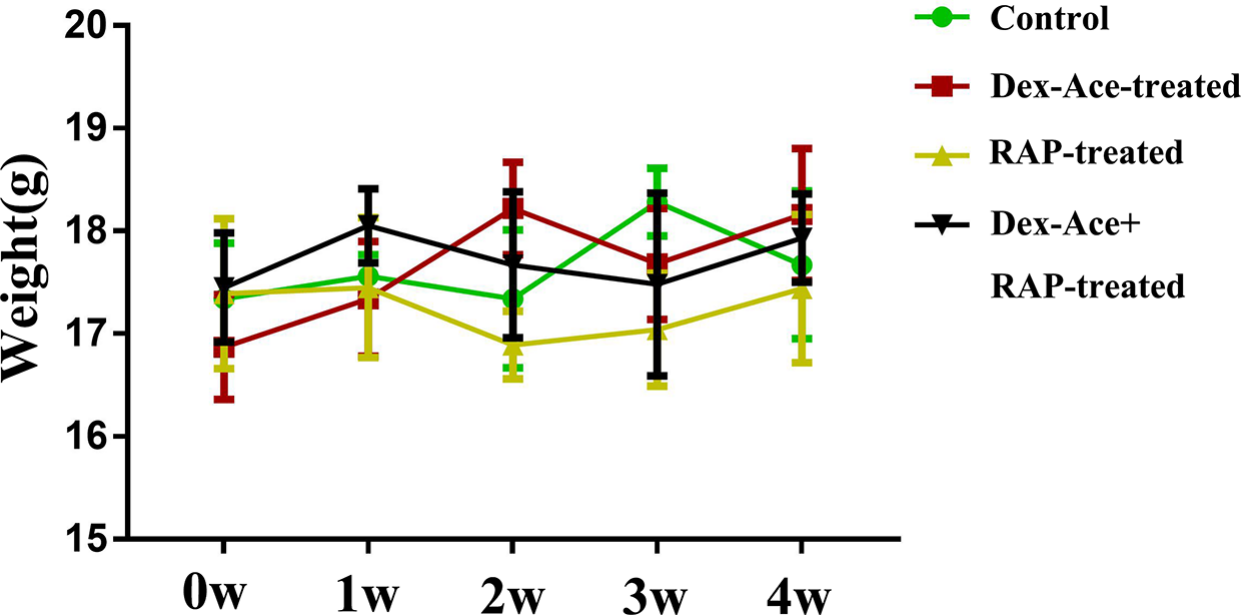
At 0–4 weeks, compared with the other three groups, there was no significant change and fluctuation in the body weight of the Dex-Ace+RAP-treated group. (n = 20; *p* > 0.05).

In order to evaluate the effect of Dex-Ace treatment and RAP treatment on the whole body of mice, we selected mice with no statistically significant difference (P> 0.05) in initial body weight comparison. After different treatments, we measured the body weight of mice weekly, and chosen body weight at the end of 0 week, 1 week, 2 week, 3 week, and 4 week for statistical analysis. There was no statistically significant difference between the body weight of each group at 1–4 weeks with its initial weight (P> 0.05). And no significant difference was seen in body weight comparison between the four groups at 1–4 weeks (P> 0.05) (**Figure 2**).

### RAP Protected RGCs From Damages by Dex-Ace Treatment

**Figure 3:**
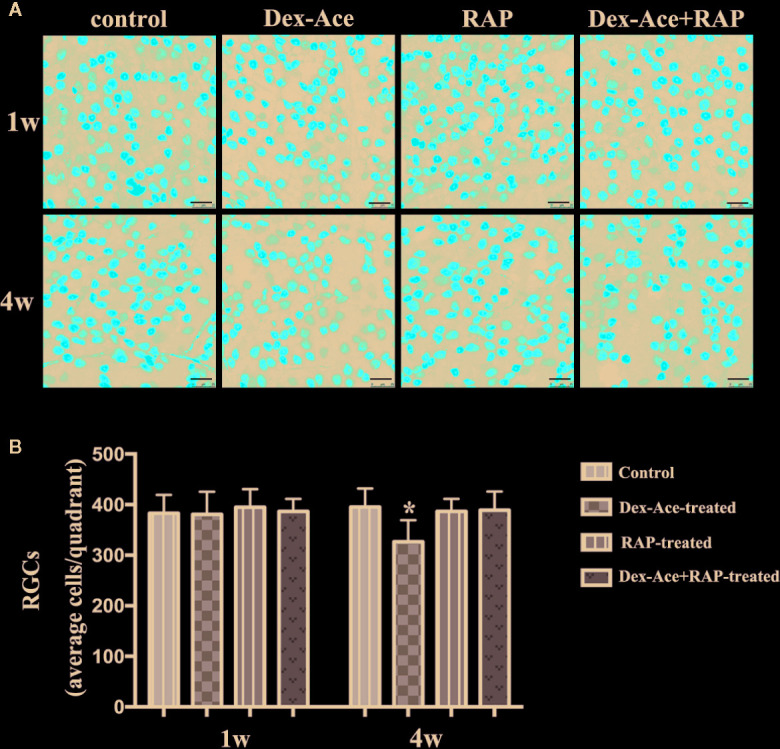
RAP increased Dex-Ace-induced reduction of RGCs at 4 weeks. **(A)** Representative BRN3a staining at 1 and 4 weeks. **(B)** Quantification of RGC survival in average four quadrants. There was no statistical difference of RGC number between groups at 1 week (*p* > 0.05). Compared with the other three groups, the RGCs were significantly reduced in the Dex-Ace group at 4 weeks (scale bar: 25 μm; n = 5; *, *p* < 0.05).

Our previous researches suggested that IOP elevation caused by Dex-Ace treatment in mice resulted in RGC loss by BRN3a immunostaining (Zeng et al., 2019). In this study, no significant difference was seen in the number of RGCs in the 4 groups of mice at 1 week. At 4 weeks, the number of RGCs in the Dex-Ace-treatment group was significantly decreased (*p* = 0.023), but not in the other three groups. Compared with the Dex-Ace-treated group (326.38 ± 42.86), Dex-Ace+RAP-treated eyes maintained normal RGC numbers in 4 weeks after treatment (388.87 ± 37.25, **Figure 3**).

### RAP Recovered Dex-Ace-Induced Ultrastructural and Histological Changes of the TM Cells

**Figure 4:**
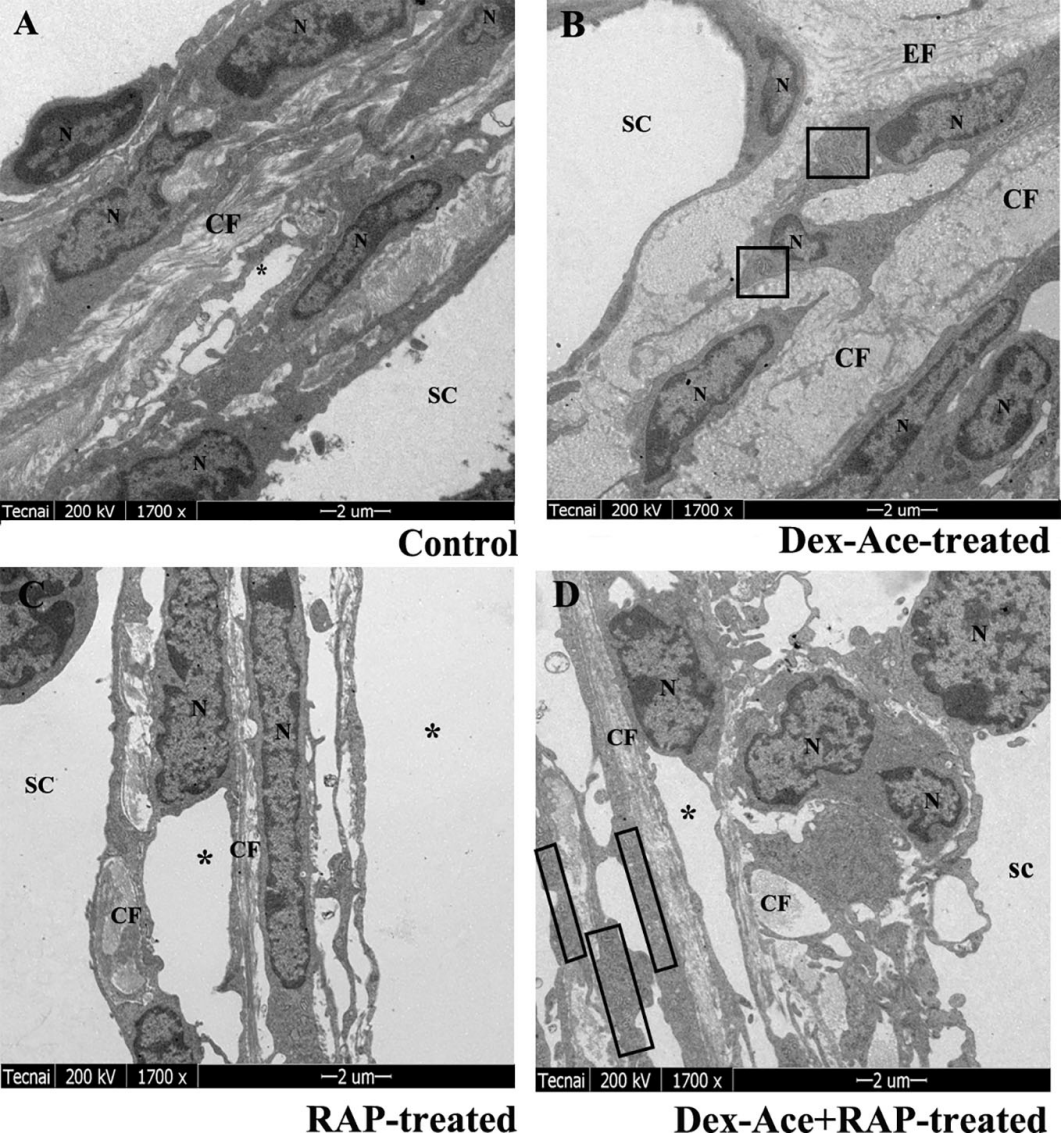
RAP normalized Dex-Ace-induced ultrastructural changes of trabecular tissues at 4 weeks. In the control and RAP-treated group **(A**, **C)**, the cell membrane was intact, the nuclear staining was uniform, and the normal mitochondria were arranged in a bundle of collagen fibers. In the Dex-Ace-treated group **(B)** the trabecular space was not obvious. The cytoplasm showed abnormal swelling of the mitochondria, increased and disorderly arranged elastic fibers, thicker, and denser outer sheath of the elastic fibers. In the Dex-Ace+RAP-treated group **(D)**, abnormal swelling of the mitochondria were also showed in the cytoplasm, but the disorderly arranged elastic fibers didn’t been found. (*: trabecular mesh gap; N, nucleus; CF, collagen fiber; EF, elastic fibers; boxes indicate swollen mitochondria; n=5; magnification; 1,700×).

Dex treatment led to many ultrastructural and histological changes in the TM cells, such as rougher cell membrane edge, poor integrity of the cell membrane, increased bundle-like collagen fibers, and inconspicuous trabecular space. Abnormal mitochondria could also be observed in the cytoplasm. Elastic fibers were increased and disordered, and the outer sheath of the elastic fibers was thick and dense (**Figure 4B**). However, synchronous RAP treatment maintained TM cells a normal morphology with no significant difference compared to the control and RAP-treated groups. The cell membrane of TM cells was smooth and intact, the nuclear staining was uniform, and the basement membrane was relatively intact and continuous. The elastic fibers were surrounded by the thin sheaths, and a large number of collagen fibers were arranged neatly (**Figures 4A–D**).

**Figure 5:**
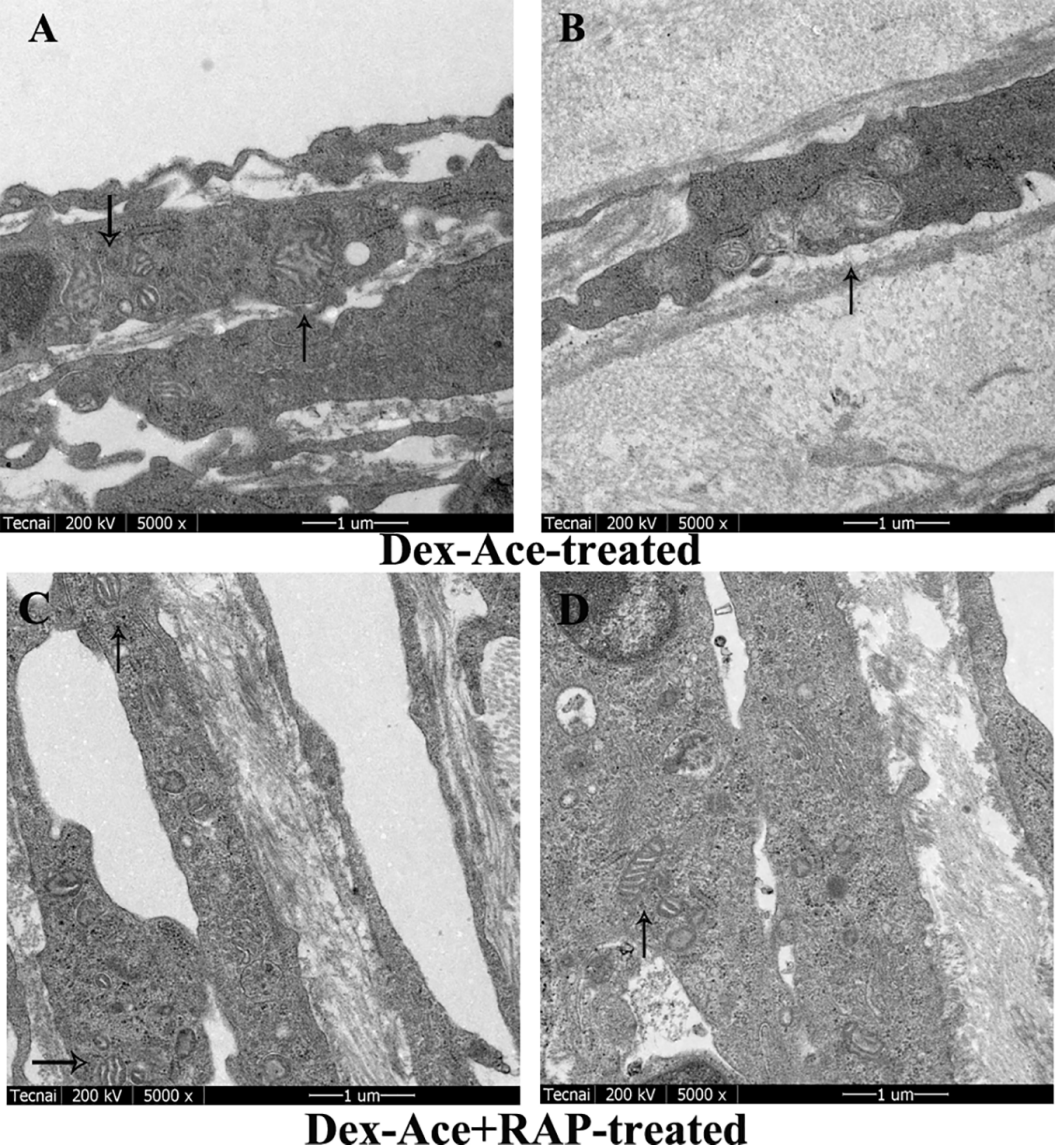
RAP normalized Dex-Ace-induced ultrastructural changes of mitochondria at 4 weeks. In the Dex-Ace-treated group **(A**, **B)**, the mitochondria were swollen and deformed, mitochondrial crests disappeared, and autophagic mitochondria fused into huge autophagosomes. In the Dex-Ace+RAP-treated group **(C**, **D)**, the mitochondria maintained normal shape and arrangement, and the mitochondrial membrane and crests were clearly visible. (arrows indicate mitochondrial; n=5; magnification: 5,000×).

In Dex-Ace-treated group, mitochondrial arrangement was disordered, the outline was blurred, the membrane was damaged and dissolved, and the crest disappeared (**Figure 5A**). In addition, it also could be observed that the mitochondria were fused and merge into giant mitochondria, which were rod-shaped and swollen (**Figure 5B**). However, synchronous RAP treatment kept the mitochondria in a normal elliptical shape, with a clear outline and crest, and a normal arrangement (**Figures 5C, D**).

### RAP Downregulated Dex-Ace-Induced α-Actin Expression

**Figure 6:**
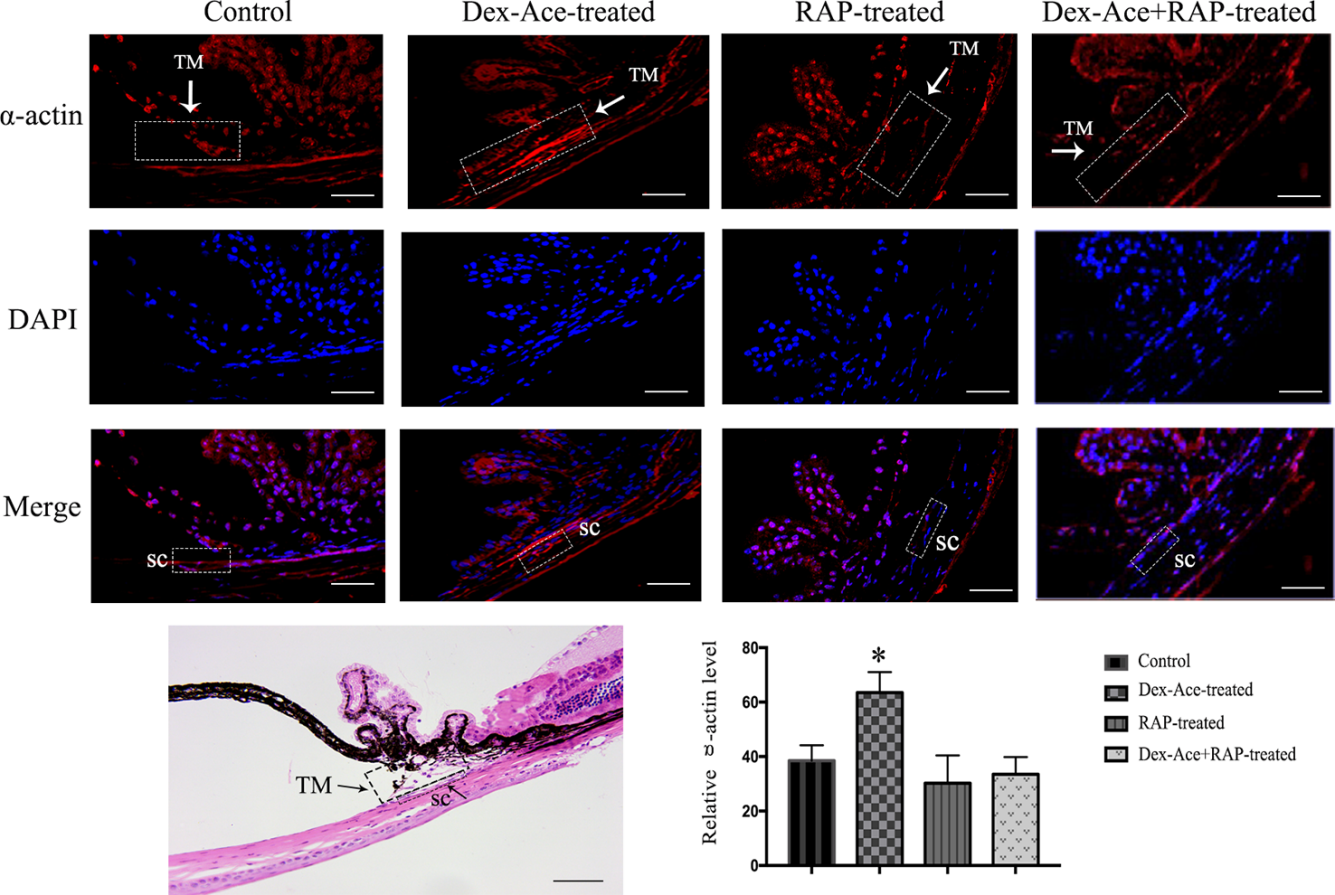
RAP decreased Dex-Ace-induced α-actin expression in mouse trabecular tissues. The fluorescence intensity of α-actin in trabecular tissues was increased in the Dex-Ace-treated group at 4 weeks. (Immunofluorescence: SC, Schlemm’s canal; magnification: 400×; scale bar: 50 μm; n = 5; *, *p* < 0.05; H&E: Magnification: 200×; scale bar: 50 mm; n = 3).

Dex-Ace treatment promoted the deposition of extracellular matrix such as fibronectin (Steely et al., 1992), collagens (Zhou et al., 1998), and α-action (Clark et al., 2005) in the trabecular tissues. To assess whether Dex-Ace treatment led to these biochemical changes in GIG mice, we experimented α-smooth muscle actin in the anterior segment tissues. Only the Dex-Ace treatment group showed obvious deposition of α-actin in the trabecular tissues. The Dex-Aced+RAP-treated group did not show an increased expression of α-actin compared to the other groups (**Figure 6**).

### RAP Upregulated TM Cell Autophagy and Downregulated Dex-Ace-Induced GIG-Related Protein Expression

**Figure 7:**
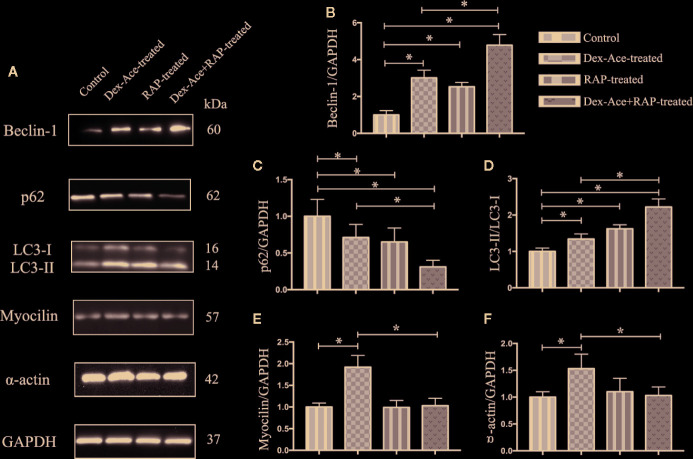
RAP enhanced Dex-Ace-reduced TM cell autophagy and Dex-Ace-induced GIG-related protein expression at the 4th week. The relative protein expression levels of Beclin-1 and LC3-II/LC3-I ratio were upregulated, and p62 was downregulated in the Dex-Ace-treated group. RAP treatment further increased beclin-1 and LC3-II/LC3-I ratio, and downregulated p62 **(A, B, C, D)**. Myocilin and a-actin were upregulated in the Dex-Ace-treated group and neutralized by RAP treatment **(A, E, F)**. (n = 6; *, *p* < 0.05).

Our previous studies suggested that numerous autophagy-related structures were discovered in the TM cells after Dex-Ace treatment (Zeng et al., 2019). Related studies demonstrated RAP increased autophagy *via* inhibiting mTOR (Hosokawa et al., 2009). To investigate whether RAP improved the autophagy in the GIG trabecular tissues, autophagy-associated proteins Beclin-1, p62 and LC3 were examined in TM cells. It has been reported that Beclin-1 was an important factor in autophagy, and its autophagic work requires adequate levels of Beclin-1 (Wirawan et al., 2012). p62, as a marker of autophagic flux, accumulates when autophagy is inhibited (Bjorkoy et al., 2009). When autophagosomes were produced, LC3-I transitioned to LC3-II, which indicated that the content of the LC3-II can laterally reflect the number of autophagosomes (Mizushima et al., 2010). Compared with the control group, the other three groups all showed a gradual increase of Beclin-1 and LC3-II/LC3-I ratio along with a decrease of p62 expression. Furthermore, Beclin-1 and LC3-II/LC3-I ratio were significantly increased and p62 was significantly decreased in the Dex-Ace+RAP-treated group compared to the Dex-Ace-treated group (**Figures 7A–D**). These results demonstrated that autophagy was activated in TM cells of GIG mice, suggesting that RAP upregulated TM cell autophagy in GIG mice.

Dex treatment also induces several biochemical modifications in the TM cells, such as enhanced accumulation of collagens, fibronectin, and α-smooth muscle actin (Patel et al., 2017). RAP downregulated α-actin expression, we next assessed the GIG-related proteins myocilin and α-actin in TM cells. Only the Dex-Ace-treated group showed increased expression of myocilin and α-actin. There was no significant difference of myocilin and α-actin expression in the other three groups (**Figures 7A, E, F**), suggesting that RAP inhibited overexpression of myocilin and α-actin after Dex-Ace treatment.

## Discussion

GIG is a secondary open angle glaucoma owing to the increased outflow resistance of the trabecular mesh water outflow channel. Many studies explored the effects of GCs on TM tissues and cells. Recently, more and more studies suggested that the autophagy homeostasis of TM cells in the glaucoma outflow pathway changed (Kitaoka et al., 2013). Our previous researches indicated that autophagy and the damaging histological changes in the TM tissues were increased in the GIG mice model. In this study, we employed this female GIG mice model to assess whether the use of Dex combined with autophagy activator RAP affected GIG progression. In female mice, hormone injection induced less stress response, such as fight and bite, resulting in less mortality rate. Our results indicated that Dex-Ace treatment induced a fast and obvious elevation of IOP, and increased α-actin and autophagy level in the TM tissues. Moreover, the loss of RGCs after DEX-Ace treatment suggested that the GIG mice model might be able to construct optic neuropathy,

Autophagy, a cellular self-digestion mechanism, is a process that catalyzes the degradation of injured organelles an protein owning to metabolism on body (Knoferle et al., 2010; Kroemer et al., 2010; Dash et al., 2019), and will further causes cell apoptosis. Induction of autophagy helps cells adapt to environmental changes by increasing the turnover of proteins and organelles, which in turn affects other cells and metabolic stress, and assist in rebalancing cell and organelle functions, suggesting that activation of autophagy may be the TM cellular original response to stress and balance (Kumari et al., 2012).

In our studies, mTOR inhibitor RAP was used as an autophagy activator to treat GIG mice. RAP was reported to bind FKBP12 and block the active site of mTOR, resulting in suppressed mTORC1 activity (Heitman J and Hall, 1991; Caron et al., 2010). GIG mice had no IOP elevation under RAP treatment, indicating that RAP-induced autophagy under Dex-Ace-treated conditions controlled IOP in mice. The number of RGCs was significantly increased in Dex-Ace+RAP-treated GIG mice, suggesting RAP protected RGCs from Dex-Ace-induced cell apoptosis, which might inhibit the release of neurotoxic mediators by modulating NF-kB signaling and directly inhibiting RGC apoptosis (Mizushima et al., 2010; Su et al., 2014). Besides, Han et al. pointed out that activated autophagy took part in RAP-mediated inhibition of BV2 microglia activation, suggesting that RAP mediated neuroprotection *via* enhancing autophagy in glaucoma (Han et al., 2013). Moreover, previous studies stated that RAP rescued RGCs *via* downregulating retinal protein REDD1 and working on the mTOR/HIF-1 pathway to vascular endothelial growth factor (VEGF) production in the photoreceptors and retinal pigment epithelial cells case (Bird, 2010).

Electron microscope observation and immunohistochemical analysis indicated that swollen and increased mitochondria accompanied by overexpressed extracellular matrix were observed in the Dex-Ace-treated mice, but not in Dex-Ace+RAP-treated mice. Damaged mitochondrial quality emphasized the impaired mitochondrial dynamics and mitophagy. Cell damage caused dynamic change in mitochondrial fission, leading to fragmental division of mitochondria and ultimately resulting in cell death (Saxena et al., 2019). Several publications reported that mitochondria affected oxidative stress (Osborne et al., 2014). It was also reported that these swollen mitochondria induced transient elevation of cytosolic calcium concentration, which in turn activated the calmodulin-dependent pathways (Kfir-Erenfeld et al., 2010). The mechanism of mitochondrial regulation by RAP is complex and multiplex. RAP might inhibit cytoplasmic mTORC1, causing a reduced hypoxia-inducible factor (HIF)-1a and glycolytic flux to elevate mitochondrial oxygen consumption simultaneously (Hudson et al., 2002). Another research showed that mTORC1 improved mitochondrial biogenesis and metabolism through transcription factors YY-1 and PGC-1a (Cunningham et al., 2007). Moreover, RAP significantly induced autophagy, and suppressed oxidative stress as well as apoptosis, possibly *via* eliminating injured mitochondria (He et al., 2019).

In our previous study, Dex-Ace treatment induced autophagy to dispose of damaged TM cells. However, increased abnormal mitochondria indicated that autophagy was insufficient to resolve GC-induced damage (Zeng et al., 2019). In our current study, the subsequent rise of Beclin-1 and LC3-II/LC3-1 ratio, together with the reduction of the autophagic substrate p62/SQSTM-1, highly suggested the outcome of an ascending autophagic flux after RAP treatment in GIG mice. Previous research stated that p62 might be involved in the neurodegenerative processes because the overexpression of p62 promoted apoptosis through increasing production of caspase-8, and the knockdown of p62 reduced human glioma cell death (Zhang et al., 2013). Further studies reported that RAP-induced autophagy inhibited axonal growth in cortical neurons and that autophagy negatively regulated axonal extension through the RhoA-ROCK pathway, resulting axonal regeneration of RGCs (Ban et al., 2013; Munemasa and Kitaoka, 2015). Myocilin in TM cells is a short-lived protein (Mizushima, 2007; Su et al., 2017). The mutation of myocilin induced a toxic gain in cellular function in the endoplasmic reticulum stress of TM cells through misfolding and abnormal amyloidosis of myocilin protein (Suntharalingam et al., 2012). In conclusion, an abnormal increase in extracellular matrix caused by Dex-Ace treatment resulted in increased IOP, dysfunctional aqueous humor outflow, TM cell death, and ultimately optic nerve damage (Jacobson et al., 2001). Therefore, enhanced autophagy, which degraded the misfolded myocilin and other increased extracellular matrix, might restore TM function and reduce pathological changes in glaucoma.

## Conclusions

In this study, the relationship between autophagy and GIG was further observed by using autophagy activators. Our results indicated that RAP ameliorated increased IOP, damaged RGCs, and TM ultrastructure changes induced by Dex-Ace. Our results further elucidated the neuroprotective function of RAP, which supported the concept that RAP was potentially therapeutic target for GIG patients.

## Data Availability Statement

All datasets presented in this study are included in the article/supplementary material.

## Ethics Statement

The animal study was reviewed and approved by Association for Research in Vision and Ophthalmology Statement of the Use of Animals in Ophthalmic and Vision Research.

## Author Contributions 

XZ designed, conducted the project. XZ and SW carried out the experiments. WZ and TZ collected and analyzed the data. JR and MK assisted with the preparation of the experiments. XZ and XC prepared this manuscript. All authors contributed to the article and approved the submitted version.

## Funding

This research was supported by National Natural Science Foundation of China (81970802).

## Conflict of Interest

The authors declare that the research was conducted in the absence of any commercial or financial relationships that could be construed as a potential conflict of interest.

## References

[B1] Ban B. K. Jun M. H. Ryu H. H. Jang D. J. Ahmad S. T. Lee J. A. (2013). Autophagy negatively regulates early axon growth in cortical neurons. Mol. Cell Biol. 33 (19), 3907–3919. 10.1128/MCB.00627-13 23918799 PMC3811863

[B2] Bird A. C. (2010). Therapeutic targets in age-related macular disease. J. Clin. Invest. 120 (9), 3033–3041. 10.1172/JCI42437 20811159 PMC2929720

[B3] Bjorkoy G. Lamark T. Pankiv S. Overvatn A. Brech A. Johansen T. (2009). Monitoring autophagic degradation of p62/sqstm1. Methods Enzymol. 452, 181–197. 10.1016/S0076-6879(08)03612-4 19200883

[B4] Caccamo A. Majumder S. Deng J. J. Bai Y. Thornton F. B. Oddo S. (2009). Rapamycin rescues tdp-43 mislocalization and the associated low molecular mass neurofilament instability. J. Biol. Chem. 284 (40), 27416–27424. 10.1074/jbc.M109.031278 19651785 PMC2785671

[B5] Caron E. Ghosh S. Matsuoka Y. Ashton-Beaucage D. Therrien M. Lemieux S. (2010). A comprehensive map of the mtor signaling network. Mol. Syst. Biol. 2010 (6), 453. 10.1038/msb.2010.108 PMC3018167 21179025

[B6] Chan D. C. (2006). Mitochondria: Dynamic organelles in disease, aging, and development. Cell 125 (7), 1241–1252. 10.1016/j.cell.2006.06.010 16814712

[B7] Clark A. F. Wordinger R. J. (2009). The role of steroids in outflow resistance. Exp. Eye Res. 88 (4), 752–759. 10.1016/j.exer.2008.10.004 18977348

[B8] Clark A. F. Brotchie D. Read A. T. Hellberg P. English-Wright S. Pang I. H. (2005). Dexamethasone alters f-actin architecture and promotes cross-linked actin network formation in human trabecular meshwork tissue. Cell Motil. Cytoskeleton. 60 (2), 83–95. 10.1002/cm.20049 15593281

[B9] Cunningham J. T. Rodgers J. T. Arlow D. H. Vazquez F. Mootha V. K. Puigserver P. (2007). Mtor controls mitochondrial oxidative function through a yy1–pgc-1α transcriptional complex. Nature 450 (7170), 736–740. 10.1038/nature06322 18046414

[B10] Dash S. Aydin Y. Wu T. (2019). Integrated stress response in hepatitis c promotes nrf2-related chaperone-mediated autophagy: A novel mechanism for host-microbe survival and hcc development in liver cirrhosis. Semin. Cell Dev. Biol. 101, 20–35. 10.1016/j.semcdb.2019.07.015 31386899 PMC7007355

[B11] Deng S. Wang M. Yan Z. Tian Z. Chen H. Yang X. (2013). Autophagy in retinal ganglion cells in a rhesus monkey chronic hypertensive glaucoma model. PloS One 8 (10), e77100. 10.1371/journal.pone.0077100 24143204 PMC3797129

[B12] Eisenberg-Lerner A. Bialik S. Simon H. U. Kimchi A. (2009). Life and death partners: Apoptosis, autophagy and the cross-talk between them. Cell Death Differ. 16 (7), 966–975. 10.1038/cdd.2009.33 19325568

[B13] Eskelinen E. L. Saftig P. (2009). Autophagy: A lysosomal degradation pathway with a central role in health and disease. Biochim. Biophys. Acta 1793 (4), 664–673. 10.1016/j.bbamcr.2008.07.014 18706940

[B14] Faralli J. A. Dimeo K. D. Trane R. M. Peters D. (2018). Absence of a secondary glucocorticoid response in c57bl/6j mice treated with topical dexamethasone. PloS One 13 (3), e0192665. 10.1371/journal.pone.0192665 29499052 PMC5834162

[B15] Fini M. E. Schwartz S. G. Gao X. Jeong S. Patel N. Itakura T. (2017). Steroid-induced ocular hypertension/glaucoma: Focus on pharmacogenomics and implications for precision medicine. Prog. Retin. Eye Res. 56, 58–83. 10.1016/j.preteyeres.2016.09.003 27666015 PMC5237612

[B16] Fu Z. Löfqvist C. A. Liegl R. Wang Z. Sun Y. Gong Y. (2018a). Photoreceptor glucose metabolism determines normal retinal vascular growth. EMBO Mol. Med. 10 (1), 76–90. 10.15252/emmm.201707966 29180355 PMC5760850

[B17] Fu Z. Wang Z. Liu C. H. Gong Y. Cakir B. Liegl R. (2018b). Fibroblast growth factor 21 protects photoreceptor function in type 1 diabetic mice. Diabetes 67 (5), 974–985. 10.2337/db17-0830 29487115 PMC5909994

[B18] Gong Y. Fu Z. Edin M. L. Liu C. Wang Z. Shao Z. (2016a). Cytochrome p450 oxidase 2c inhibition adds to omega-3 long-chain polyunsaturated fatty acids protection against retinal and choroidal neovascularization. Arterioscler. Thromb. Vasc. Biol. 36 (9), 1919–1927. 10.1161/ATVBAHA.116.307558 27417579 PMC5010176

[B19] Gong Y. Shao Z. Fu Z. Edin M. L. Sun Y. Liegl Y. (2016b). Fenofibrate inhibits cytochrome p450 epoxygenase 2c activity to suppress pathological ocular angiogenesis. EBioMedicine 13, 201–211. 10.1016/j.ebiom.2016.09.025 27720395 PMC5264653

[B20] Han H. E. Kim T. K. Son H. J. Park W. J. Han P. L. (2013). Activation of autophagy pathway suppresses the expression of inos, il6 and cell death of lps-stimulated microglia cells. Biomol. Ther. (Seoul) 21 (1), 21–28. 10.4062/biomolther.2012.089 24009854 PMC3762303

[B21] He J. N. Zhang S. D. Qu Y. Wang H. L. Tham C. C. Pang C. P. (2019). Rapamycin removes damaged mitochondria and protects human trabecular meshwork (tm-1) cells from chronic oxidative stress. Mol. Neurobiol. 56 (9), 6586–6593. 10.1007/s12035-019-1559-5 30903531

[B22] Heitman J M. N. Hall M. N. (1991). Targets for cell cycle arrest by the immunosuppressant rapamycin in yeast. Science 253, 905–909. 10.1126/science.1715094 1715094

[B23] Hirt J. Liton P. B. (2017). Autophagy and mechanotransduction in outflow pathway cells. Exp. Eye Res. 158, 146–153. 10.1016/j.exer.2016.06.021 27373974 PMC5199638

[B24] Hosokawa N. Hara T. Kaizuka T. Kishi C. Takamura A. Miura Y. (2009). Nutrient-dependent mtorc1 association with the ulk1-atg13-fip200 complex required for autophagy. Mol. Biol. Cell 20 (7), 1981–1991. 10.1091/mbc.e08-12-1248 19211835 PMC2663915

[B25] Hudson C. C. Liu M. Chiang G. G. Otterness D. M. Loomis D. C. Kaper F. (2002). Regulation of hypoxia-inducible factor 1 expression and function by the mammalian target of rapamycin. Mol. Cell. Biol. 22 (20), 7004–7014. 10.1128/MCB.22.20.7004-7014.2002 12242281 PMC139825

[B26] Jacobson N. Andrews M. Shepard A. R. Nishimura D. Searby C. Fingert J. H. (2001). Non-secretion of mutant proteins of the glaucoma gene myocilin in cultured trabecular meshwork cells and in aqueous humor. Hum. Mol. Genet. 10 (2), 117–125. 10.1093/hmg/10.2.117 11152659

[B27] Kasetti R. B. Phan T. N. Millar J. C. Zode G. S. (2016). Expression of mutant myocilin induces abnormal intracellular accumulation of selected extracellular matrix proteins in the trabecular meshwork. Invest. Ophthalmol. Visual Sci. 57 (14), 6058–6069. 10.1167/iovs.16-19610 27820874 PMC5102566

[B28] Kfir-Erenfeld S. Sionov R. V. Spokoini R. Cohen O. Yefenof E. (2010). Protein kinase networks regulating glucocorticoid-induced apoptosis of hematopoietic cancer cells: Fundamental aspects and practical considerations. Leuk. Lymphoma 51 (11), 1968–2005. 10.3109/10428194.2010.506570 20849387

[B29] Kitaoka Y. Munemasa Y. Kojima K. Hirano A. Ueno S. Takagi H. (2013). Axonal protection by nmnat3 overexpression with involvement of autophagy in optic nerve degeneration. Cell Death Dis. 4, e860. 10.1038/cddis.2013.391 24136224 PMC3920931

[B30] Knoferle J. Koch J. C. Ostendorf T. Michel U. Planchamp V. Vutova P. (2010). Mechanisms of acute axonal degeneration in the optic nerve in vivo. Proc. Natl. Acad. Sci. U.S.A. 107 (13), 6064–6069. 10.1073/pnas.0909794107 20231460 PMC2851885

[B31] Kroemer G. Marino G. Levine B. (2010). Autophagy and the integrated stress response. Mol. Cell 40 (2), 280–293. 10.1016/j.molcel.2010.09.023 20965422 PMC3127250

[B32] Kumari S. Mehta S. L. Li P. A. (2012). Glutamate induces mitochondrial dynamic imbalance and autophagy activation: Preventive effects of selenium. PloS One 7 (6), e39382. 10.1371/journal.pone.0039382 22724008 PMC3378533

[B33] Kwon Y. H. Fingert J. H. Kuehn M. H. Alward W. L. (2009). Primary open-angle glaucoma. N Engl. J. Med. 360 (11), 1113–1124. 10.1056/NEJMra0804630 19279343 PMC3700399

[B34] Levine B. Klionsky D. J. (2004). Development by self-digestion: Molecular mechanisms and biological functions of autophagy. Dev. Cell 6 (4), 463–477. 10.1016/S1534-5807(04)00099-1 15068787

[B35] Li J. Liu C. H. Sun Y. Gong Y. Fu Z. Evans L. P. (2014). Endothelial twist1 promotes pathological ocular angiogenesis. Invest. Ophthalmol. Visual Sci. 55 (12), 8267–8277. 10.1167/iovs.14-15623 25414194 PMC4541480

[B36] Malagelada C. Jin Z. H. Jackson-Lewis V. Przedborski S. Greene L. A. (2010). Rapamycin protects against neuron death in in vitro and in vivo models of parkinson’s disease. J. Neurosci. 30 (3), 1166–1175. 10.1523/JNEUROSCI.3944-09.2010 20089925 PMC2880868

[B37] Mizushima N. Yoshimori T. Levine B. (2010). Methods in mammalian autophagy research. Cell 140 (3), 313–326. 10.1016/j.cell.2010.01.028 20144757 PMC2852113

[B38] Mizushima N. (2007). Autophagy: Process and function. Genes Dev. 21 (22), 2861–2873. 10.1101/gad.1599207 18006683

[B39] Munemasa Y. Kitaoka Y. (2015). Autophagy in axonal degeneration in glaucomatous optic neuropathy. Prog. Retin. Eye Res. 47, 1–18. 10.1016/j.preteyeres.2015.03.002 25816798

[B40] Osborne N. N. Álvarez C. N. del Olmo Aguado S. (2014). Targeting mitochondrial dysfunction as in aging and glaucoma. Drug Discovery Today 19 (10), 1613–1622. 10.1016/j.drudis.2014.05.010 24880106

[B41] Overby D. R. Bertrand J. Tektas O. Y. Boussommier-Calleja A. Schicht M. Ethier C. R. (2014). Ultrastructural changes associated with dexamethasone-induced ocular hypertension in mice. Invest. Ophthalmol. Visual Sci. 55 (8), 4922–4933. 10.1167/iovs.14-14429 25028360 PMC4126794

[B42] Patel G. C. Phan T. N. Maddineni P. Kasetti R. B. Millar J. C. Clark A. F. (2017). Dexamethasone-induced ocular hypertension in mice. Am. J. Pathol. 187 (4), 713–723. 10.1016/j.ajpath.2016.12.003 28167045 PMC5397678

[B43] Porter K. M. Jeyabalan N. Liton P. B. (2014). Mtor-independent induction of autophagy in trabecular meshwork cells subjected to biaxial stretch. Biochim. Biophys. Acta 1843 (6), 1054–1062. 10.1016/j.bbamcr.2014.02.010 24583119 PMC3988584

[B44] Prevel N. Allenbach Y. Klatzmann D. Salomon B. Benveniste O. (2013). Beneficial role of rapamycin in experimental autoimmune myositis. PloS One 8 (11), e74450. 10.1371/journal.pone.0074450 24265670 PMC3827074

[B45] Saxena S. Mathur A. Kakkar P. (2019). Critical role of mitochondrial dysfunction and impaired mitophagy in diabetic nephropathy. J. Cell. Physiol. 234 (11), 19223–19236. 10.1002/jcp.28712 31032918

[B46] Steely H. T. Browder S. L. Julian M. B. Miggans S. T. Wilson K. L. Clark A. F. (1992). The effects of dexamethasone on fibronectin expression in cultured human trabecular meshwork cells. Invest. Ophthalmol. Visual Sci. 33 (7), 2242–2250. 1607235

[B47] Ster A. M. Popp R. A. Petrisor F. M. Stan C. Pop V. I. (2014). The role of oxidative stress and vascular insufficiency in primary open angle glaucoma. Clujul. Med. 87 (3), 143–146. 10.15386/cjmed-295 26528013 PMC4508597

[B48] Su W. Li Z. Jia Y. Zhuo Y. (2014). Rapamycin is neuroprotective in a rat chronic hypertensive glaucoma model. PloS One 9 (6), e99719. 10.1371/journal.pone.0099719 24923557 PMC4055719

[B49] Su Y. Wu J. He J. Liu X. Chen X. Ding Y. (2017). High insulin impaired ovarian function in early pregnant mice and the role of autophagy in this process. Endocr. J. 64 (6), 613–621. 10.1507/endocrj.EJ16-0494 28420820

[B50] Suntharalingam A. Abisambra J. F. O’Leary J. C. Koren J. Zhang B. Joe M. K. (2012). Glucose-regulated protein 94 triage of mutant myocilin through endoplasmic reticulum-associated degradation subverts a more efficient autophagic clearance mechanism. J. Biol. Chem. 287 (48), 40661–40669. 10.1074/jbc.M112.384800 23035116 PMC3504779

[B51] Vidal-Sanz M. Valiente-Soriano F. J. Ortín-Martínez A. Nadal-Nicolás F. M. Jiménez-López M. Salinas-Navarro M. (2015). Retinal neurodegeneration in experimental glaucoma. Prog. Brain. Res. 220, 1–35. 26497783 10.1016/bs.pbr.2015.04.008

[B52] Wang W. H. Millar J. C. Pang I. H. Wax M. B. Clark A. F. (2005). Noninvasive measurement of rodent intraocular pressure with a rebound tonometer. Invest. Ophthalmol. Visual Sci. 46 (12), 4617–4621. 10.1167/iovs.05-0781 16303957

[B53] Wang Z. Liu C. H. Sun Y. Gong Y. Favazza T. L. Morss P. C. (2016). Pharmacologic activation of wnt signaling by lithium normalizes retinal vasculature in a murine model of familial exudative vitreoretinopathy. Am. J. Pathol. 186 (10), 2588–2600. 10.1016/j.ajpath.2016.06.015 27524797 PMC5222984

[B54] Wirawan E. Lippens S. Vanden Berghe T. Romagnoli A. Fimia G. M. Piacentini M. (2012). Beclin1: A role in membrane dynamics and beyond. Autophagy 8 (1), 6–17. 10.4161/auto.8.1.16645 22170155

[B55] Zeng W. Wang W. Wu S. Zhu X. Zheng T. Chen X. (2019). Mitochondria and autophagy dysfunction in glucocorticoid-induced ocular hypertension/glaucoma mice model. Curr. Eye Res. 45 (2), 190–198. 10.1080/02713683.2019.1657462 31425668

[B56] Zhang Y. B. Gong J. L. Xing T. Y. Zheng S. P. Ding W. (2013). Autophagy protein p62/sqstm1 is involved in hamlet-induced cell death by modulating apotosis in u87mg cells. Cell Death Dis. 4, e550. 10.1038/cddis.2013.77 23519119 PMC3615731

[B57] Zhou L. Li Y. Yue B. Y. (1998). Glucocorticoid effects on extracellular matrix proteins and integrins in bovine trabecular meshwork cells in relation to glaucoma. Int. J. Mol. Med. 1 (2), 339–346. 10.3892/ijmm.1.2.339 9852235

[B58] Zhu H. Zhang Y. (2018). Life and death partners in post-pci restenosis: Apoptosis, autophagy, and the cross-talk between them. Curr. Drug Targets 19 (9), 1003–1008. 10.2174/1389450117666160625072521 27358059

[B59] Zode G. S. Sharma A. B. Lin X. Searby C. C. Bugge K. Kim G. H. (2014). Ocular-specific er stress reduction rescues glaucoma in murine glucocorticoid-induced glaucoma. J. Clin. Invest. 124 (5), 1956–1965. 10.1172/JCI69774 24691439 PMC4001532

[B60] Zode G. S. Kuehn M. H. Nishimura D. Y. Searby C. C. Mohan K. Grozdanic S. D. (2015). Reduction of er stress via a chemical chaperone prevents disease phenotypes in a mouse model of primary open angle glaucoma. J. Clin. Invest. 125 (8), 3303. 10.1172/JCI82799 PMC4563763 26237042

